# Urban heat indicator map for climate adaptation planning

**DOI:** 10.1007/s11027-015-9669-5

**Published:** 2015-07-22

**Authors:** M. A. M. de Groot-Reichwein, R. J. A. van Lammeren, H. Goosen, A. Koekoek, A. K. Bregt, P. Vellinga

**Affiliations:** 1 Wageningen University and Research Centre, Wageningen, The Netherlands; 2 Geodan BV, Amsterdam, The Netherlands; 3 WUR-Alterra, P.O. Box 47, 6700 AA Wageningen, The Netherlands

**Keywords:** Information enrichment chain, Urban heat, Visualisation, Land use change, Climate change

## Abstract

By 2050, 75 % of the world’s population will live in cities and the occurrence of heat wave events might have doubled. Mapping the climate and land use change impact for urban heat events should set the agenda for adaptation planning at the local scale. Literature on urban heat mapping does not reveal a clear indicator to visualise the urban heat impacts that includes consequences of land use and climate changes for planning purposes. This paper introduces a stepwise approach to develop a single complex indicator to map the urban heat impact for local climate adaptation planning processes. Information on climatic drivers and land use characteristics are combined and projected for future land use and climate change impacts. Next, several visualisation techniques are developed to investigate which techniques are most effective to visualise complex information with multiple variables in one visualisation. A usability test is performed to investigate how indicator and map meet the information and communication needs of policy makers. Our findings reveal that it is important to add information on future impacts to set the agenda for adaptation planning at the local scale. Applying cartographic techniques in a map series presentation has proven to be effective to map complex information in a single image and fulfil most of the identified information needs. Based on our finding, we introduce the information enrichment chain as a promising approach to support local adaptation planning.

## Introduction

An urban heat island (UHI) is the phenomenon that occurs when the temperature in an urban area is significantly higher than in its surrounding areas. The UHI effect can be related to urban characteristics like impervious surface and green fraction and is most noticeable during nights (Oke 1978). The UHI effect is affected by both climate and land use changes (Bulkeley 2013). Temperature rise (as a result of global warming) and further urbanisation will increase the frequency and duration of heat wave events and might result in enhanced mortality and less labour activity (Luber and McGeehin 2008). However, the relation between global warming and the UHI effect is hardly addressed in scientific literature (Alcoforado and Andrade 2008). Differences in temporal and spatial scales, research methods and data sets are given as main reason. Spatial planning as a multi-level system might overcome this problem by cascading down the spatial impacts from global to local (Wilson 2006). Translating and tailoring global impact information into policy-relevant and usable science helps to set the agenda for adaptation at the local scale (WMO 2011). Local adaption contributes to global adaptation by taking climate change impacts into account in the location and design of development (Yow 2007).

Goosen et al. (2013) already emphasise the need of information visualisation to support the local spatial planning processes were much of the adaptation took place. Information visualisation aims at the visual representations of abstract data to support human cognition (Horn 1999). It comprises various visualisation techniques like charts, diagrams, graphs, tables and maps (Tufte 1990). Various authors (Burch et al. 2010; Shaw et al. 2009; Sheppard 2005; Sheppard and Cizek 2009; Sheppard et al. 2008, 2011) investigated the role of landscape visualisation to stimulate adaptation behaviour. Landscape visualisations are highly realistic three dimensional (3D) representations of actual places and on-the-ground conditions. Although landscape visualisations proved to be effective planning tools, it is recognised that other more conventional visualisation may be needed in addition (Sheppard 2005). We assume that using an adequate cartographic presentation supports the information exchange of climate impact for planning purposes. Cartography is the science and practice of creating and understanding maps (Morrison 1974). Maps have proven to be powerful tools in the process of spatial planning as planners and practitioners are familiar with cartographic materials (Al-Kodmany 2002). However, climate impact information is still hard to catch by useful indicators (Hinkel 2011) and the effectiveness and efficiency of different mapping techniques have rarely been tested with users (Garlandini and Fabrikant 2009).

This paper aims at translating scientific information on urban heat into policy-relevant and usable information to support local adaptation planning processes. The research questions addressed in this paper are the following:

I. What policy-relevant urban heat indicator for local climate adaptation planning processes can be constructed?
II. How can such an urban heat indicator be visualised?

To address the research questions and reveal a policy-relevant urban heat indicator and visualisation technique, we first present a literature review and analysis on UHI mapping. Next, we describe the different steps taken to construct a policy-relevant urban heat indicator, followed by a section describing the design of a map that meets the information and communication needs of policy makers. We conclude with a reflection on our methodology and an overall conclusion.

## Literature review on urban heat mapping

**Fig. 1:**
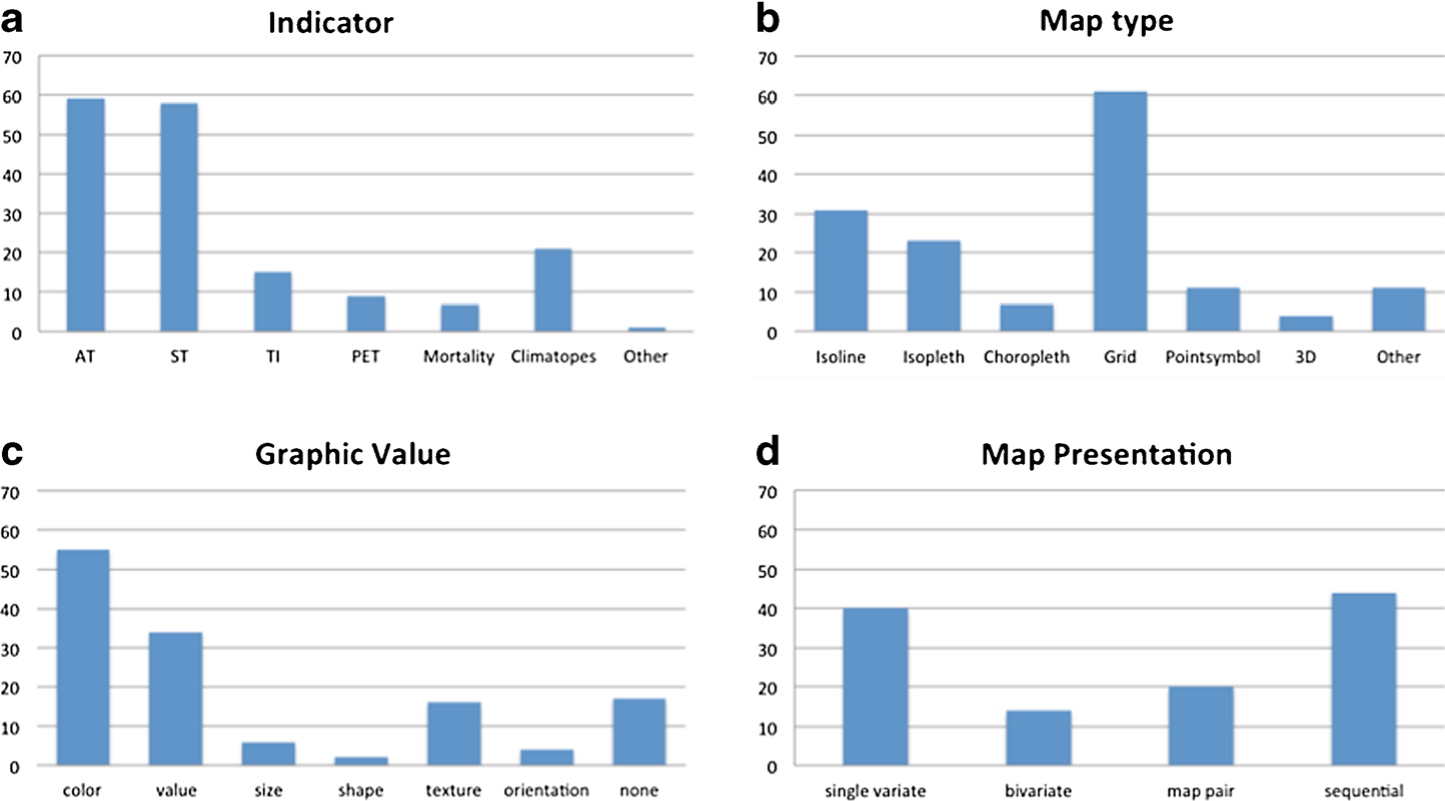
Results of analysis on urban heat island (UHI) mapping literature. One hundred thirty-three articles containing a map, revealing the UHI were analysed for **a** indicator used: air temperature (AT), surface temperature (ST), thermal inertia (TI), physical equivalent temperature (PET), mortality, climatopes or other; **b** map type: isoline, isopleth, choropleth, grid, point symbol, 3D or other; **c** graphic variable: colour hue, value, size, texture, orientation or none; **d** map presentation: single variate, bivariate, map pair or sequential presentation

More than 250 scientific articles on urban heat mapping indicators and mapping techniques have been studied. One hundred thirty-three articles did use a cartographic map to indicate the phenomenon of urban heat. These articles were further analysed by (1) method of data measurement, (2) nature of indicator, (3) mapping technique (map type, graphic variable and map presentation), (4) involvement of policy makers and (5) future projection of climate or land use change (Fig. 1). The majority of the articles used air and surface temperature (sometimes combined) to present the phenomenon. A grid map was the most common map since satellite images were used to present surface temperature or thermal inertia differences. Isolines were commonly used for air temperature measurements. Colour hue was the most popular graphic variable but mostly used for quantitative data instead of qualitative data. Map series were used to present the phenomenon for different time steps (e.g. differences between day and night, season or past land use changes). Based on these analyses, we conclude that there is no common indicator and mapping technique to reveal the urban heat impact for planning purposes. Only a few maps include information of land use change or climate changes impacts. In none of the case studies, indicators nor mapping techniques were systematically evaluated on their relevance for policy makers.

## Urban heat indicator

**Fig. 2:**
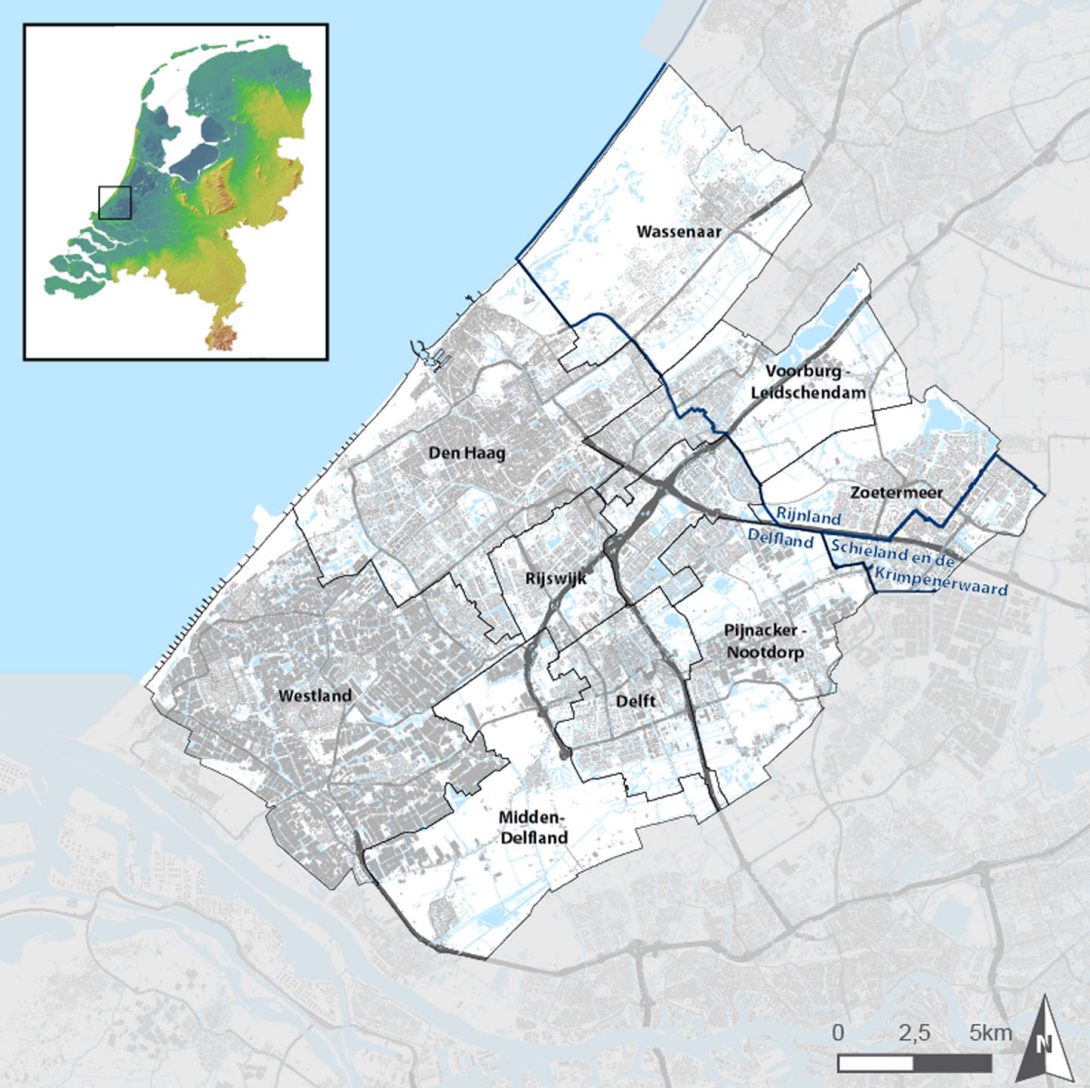
Case study area: Haaglanden region. The Haaglanden region is located in the south-western part of the Netherlands and comprises The Hague conurbation and a substantial concentration of greenhouse horticulture bordering on the North Sea. Land use is very intensive: the built-up area covers 40 % of the area and another 30 % is used by greenhouse. The region has to accommodate economic and population growth objectives, which could make the area even more vulnerable for the effects of climate change

The development of the urban heat indicator took place in close cooperation with policy makers of the Haaglanden region during the UHI project. The Haaglanden region (see Fig. 2) is one of the hotspots, within the Dutch Knowledge for Climate research program. The nine Haaglanden municipalities, the two water boards and the provincial authority took the initiative to set up a regional adaptation strategy (RAS). Urban heat was indicated as one of the most serious possible threats for the region as the built-up area covers 40 % of the area and another 30 % is used by greenhouse. The region still entails major spatial challenges in order to continue accommodating economic growth and the increase in population, making the region even more vulnerable for the effects of climate change.

**Fig. 3:**
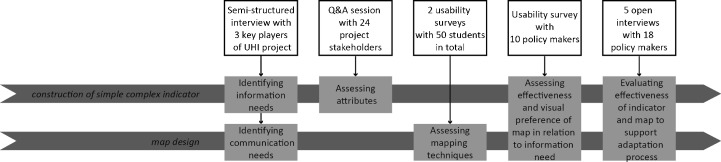
Overview of the evaluation moments with the stakeholders and students to construct and evaluate both indicator and map presentation

Figure 3 gives an overview of the interview, questionnaire, surveys and project evaluations we have organised with the stakeholders and students to investigate how indicator and map meet the information and communication needs of policy makers.

At the start of the UHI project, we held semi-structured interviews with three key players of the RAS. Topics discussed during the interview varied from aim, planning phase, target group, to level of scale and detail, outcome and complexity of the project (e.g. amount of stakeholders involved, conflicting interests or interrelations with other policy processes). A distinction was made between information needs and communication needs. Identifying information needs was necessary to determine the core variables of interest and urban heat indicators to discuss effects and sensitivity. Questions that were answered are which effect, variables and scenarios should be visualised at which level of scale? Effects of heat for example can be indicated by the air temperature during nights, but also by human comfort or surface temperature. Identifying communication needs centred the message, goal and outcome of a visualisation. Is the visualisation meant to inspire or to underpin the policy-making process?

**Fig. 4:**
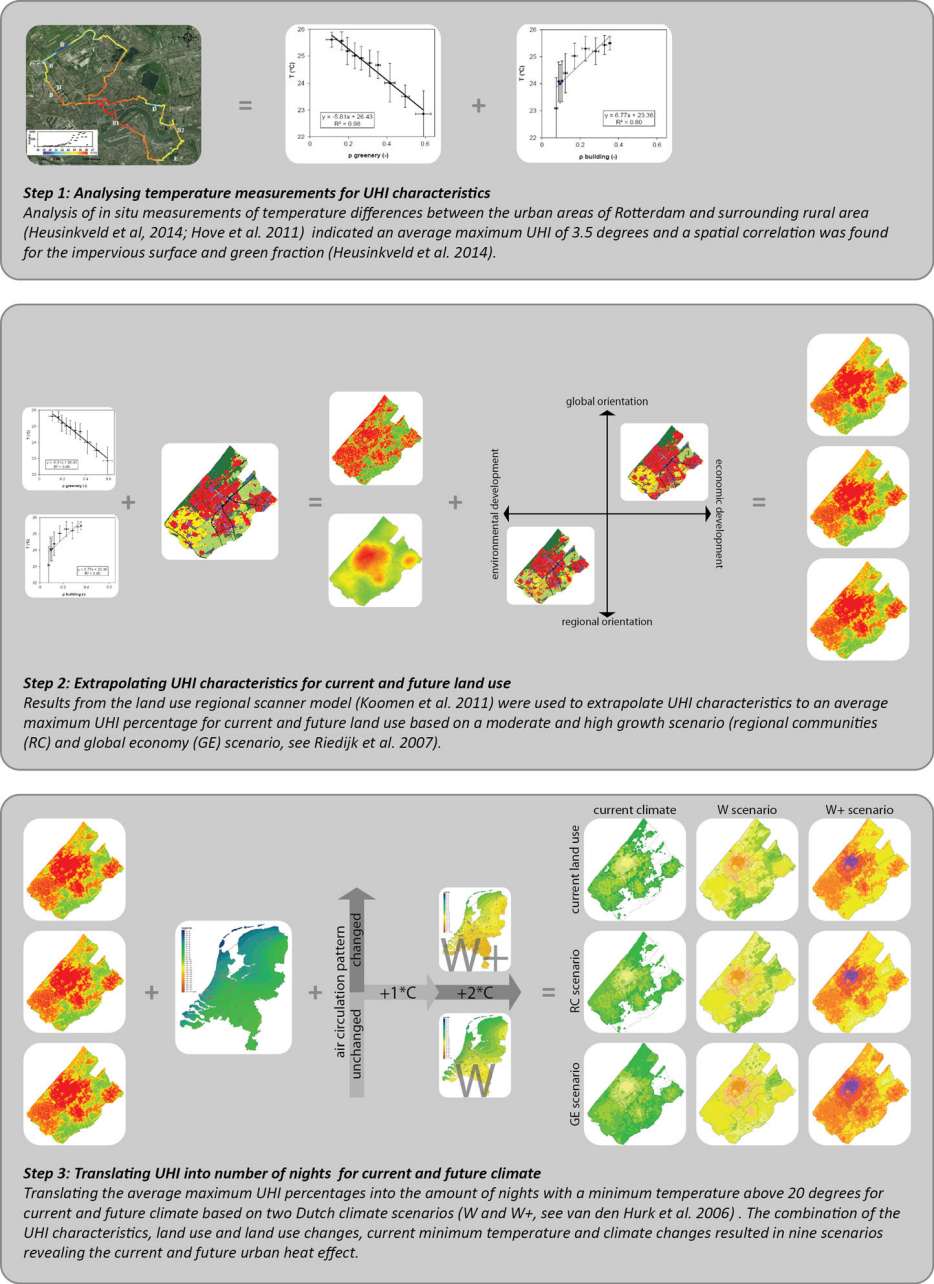
Data transformation chain. A data transformation was performed to construct the urban heat indicator for the Haaglanden region. The chain consists of three main steps in which urban heat island (UHI) measurements were used to indicate UHI characteristics (step 1). Subsequently, the characteristics were extrapolated to an UHI percentage for current and future land use (step 2) and finally translated to the amount of nights per year with a minimum temperature above 20° for current and future climate

Based on the information needs, the different elements of the indicator were constructed. We defined an indicator as a function of variables, providing an indication, i.e. an entity that can be used as an argument of a function used to take a decision (Riley 2001). A variable then was defined as an observable characteristic of an object or event that can be measured. Nocturnal air temperature and UHI characteristics were chosen as variables to develop an urban heat indicator. To provide an indication of future urban heat island patterns for the Haaglanden region, we combined and transformed information from various sources. This approach is meant to provide a quick assessment of potential changes based on currently available knowledge. The different steps of our data transformation are described in the next sections and illustrated in Fig. 4.

### Step 1: analysing temperature measurements for urban heat island characteristics

In situ measurements of temperature differences between the urban areas of Rotterdam and surrounding rural area (Heusinkveld et al. 2014; Hove et al. 2011) were analysed. These analyses indicated an average maximum UHI of 3.5° and a spatial correlation was found for the impervious surface and green fraction (Heusinkveld et al. 2014).

### Step 2: extrapolating UHI characteristics into UHI percentage for current and future land use

The UHI characteristics were extrapolated for the Haaglanden region using representations of current land use taken from the Land Use Scanner model (Riedijk et al. 2007). The extrapolation resulted in an average maximum UHI percentage for each 250 by 250 m grid cell. To reveal future impacts of urbanisation, existing scenario-based simulations of land use change were applied. These simulations depict two diverging social economic scenarios (regional communities and global economy) that were developed by CPB and RPB (2006) and that offer the basis for many Dutch assessment studies of future changes. The land use simulations were created with the land use regional scanner model (Koomen and Borsboom-van Beurden 2011).

### Step 3: translating UHI into number of nights for current and future climate

The average maximum UHI percentages were translated in the amount of nights with a minimum temperature above 20° (tropical nights). The minimum temperature was chosen as climatic driver to indicate the nocturnal UHI effect. The limit (threshold) was set at 20 °C as above that temperature thermal well-being is no longer certain (Gabriel and Endlicher 2011). Temperature measurements of meteorological stations (Sluijter 2011) were corrected with the UHI effect to determine the amount of nights with a minimum temperature above 20° for the Haaglanden region. To reveal the future impact of climate change, the number of nights were extrapolated for two Dutch climate scenarios (van den Hurk et al. 2006) using the Climate Explorer (KNMI (n.d.)). The combination of the UHI characteristics, land use and land use changes, current minimum temperature and climate changes resulted in nine scenarios revealing the current and future urban heat effect.

The constructed urban heat indicator was assessed in a multiple choice questions and answers session with 24 stakeholders of the case study area. Aim of the assessment was to measure the effectiveness, efficiency and user preference of different attributes of the urban heat indicator. The urban heat indicator was therefore presented for three different attributes, each with different number of classes: (A1) areas with the number of tropical nights (13 classes), (A2) areas with more than 10 tropical nights (9 classes) and (A3) areas of which the number of tropical nights increases compared to the current situation (6 classes). Questions were centred on three tasks: (a) depicting vulnerable area in different scenarios, (b) depicting differences between climate and land use change separately and (c) depicting the amount of nights. Answers were collected via voting buttons to measure time in relation to the correctness of the answer. Twenty-four respondents participated in the first evaluation. Fourteen of them were policy makers involved in the adaptation planning process of the case study area and thus representative for the intended user group. The other 10 respondents were working for a research institute or consultancy agency. The answers of the latter were not used for the remainder of the case study as only policy makers were indicated as end users. However, their results were quite comparable, as we will discuss below. Maps depicting the increase (A3) had the lowest score both on effectiveness/efficiency (9 out of 14 policy makers and 6 out of 10 researchers) and preference (0 out of 14 policy makers and 1 out of 10 researchers). Maps depicting the number of nights (A1) had the highest score both on effectiveness/efficiency (13 out of 14 policy makers and 10 out of 10 researchers) and preference (9 out of 14 policy makers and 8 out of 10 researchers).

In the end stage of the UHI project, a final evaluation was held via open-interviews with 18 involved policy makers. The interviews took place during five project-meetings in which the final adaptation strategy for the region was discussed. The aim of the final evaluation was to determine whether the indicator supports the adaptation planning process of the case study area. Policy makers were also asked to comment on the indicator and give suggestions for further improvement. The policy makers perceived all different components of the indicator (number of nights, land use changes and climate changes) as relevant. Especially in the phase of agenda setting, it became crucial to make a comparison between current and future situations to clearly visualise the urgency. Revealing which areas are most vulnerable set the agenda for spatial planning to include adaptation measures.

Based on both, the assessment and the evaluation, we conclude that a single complex indicator provides accessible, useful and relevant information for the adaptation planning process at the regional scale. Defining the different components of the indicator together with policy makers improves the information exchange between science and policy and provides a basis for the development of a regional adaptation strategy.

## Map design

To address our second research question, we investigated different mapping techniques to visualise the single complex indicator. Different mapping techniques exist to express multivariate data. However, to be effective, the amount of variables to combine in one map has to be limited (Bertin 1983; Geels 1987; Kraak and Ormeling 2010). The development of maps took place via an iterative research by design approach. In research by design, the design process forms a pathway through which new insights, knowledge, practices or products are developed and analysed in continuous interplay (De Jong and Van Der Voordt 2002). Aim of the research was to construct a single indicator and combine it for all scenarios in one map presentation. We first have investigated which map types and corresponding graphic variables are effective to map our urban heat indicator for a single scenario. Next, we have investigated different map presentations to visualise all nine scenarios in one visualisation.

### Map types

Kraak and Ormeling (2010) made a subdivision of map types based on measurement scale (nominal, ordinal, interval and ratio) and the nature of distribution of the object (point, line, areal or volume data). Six map types (isoline, isopleth, choropleth, point symbol, diagram and grid) were investigated. Isoline maps are used to represent a phenomenon as a continuous distribution. Each line connects points with an equal value. An isopleth is used for contour lines that depict a variable which cannot be measured at a point, but which must be interpolated from discrete data collected over an area. A stepped surface is a 3D representation of the data in which the height is made proportional to the numerical attribute of the data. In a choropleth, an average value for an area can be calculated and represented as discrete values. In our example, each area represents a neighbourhood. In a grid map, an average value for each grid cell is calculated. This value can be represented by a filled area (grid choropleth) or a point symbol (symbol grid). In our case, the land use data was modelled per grid. Temperature was mapped by interpolation.

### Graphic variables

Graphic or visual variables refer to the differences in map elements as perceived by the human eye. Bertin (1983) introduces a basic system of graphic variables: shape, size, orientation texture, colour hue and colour value. Bertin’s original six variables have been modified and expanded by various cartographers and authors, e.g. Morrison (1974, DiBiase et al. 1992; MacEachren 1994 and MacEachren et al. 1994). Graphic variables are very important elements of cartographic design. They help to make a map visually appealing and help to emphasize the purpose of the map (Dent 1990; Slocum et al. 2009). Size and colour value are commonly used for an overview of the distribution of data, while the use of colour hue makes it easier to see simultaneously all objects belonging to one class. Shape and colour hue both allow users to differentiate between objects. To reveal the geographical distribution of the phenomenon, the visual hierarchy should be identical to the hierarchy of the phenomenon. Variations in shape, orientation and colour hue can be best used to represent qualitative (nominal) variations in attribute data, while texture (ordinal), value (interval) and size (ratio) convey a correct perception of quantitative data.

### Map presentation

To present multiple variables in one map MacEachren (1992) describes three map presentations that could be used separately or in combination: (1) bivariate maps in which all variables are incorporated in the one single map (2) map pairs in which variables are presented in side-by-side maps or (3) map series in which a time line of maps is presented.

To combine all scenarios information in one bivariate map, a threshold for the amount of nights was introduced. By doing so, the message of the map changed: instead of the amount of days the phenomenon occurs, emphasis was made on areas in which the phenomenon occurs more than 10 nights a year. Three map types (isopleth, symbol and diagram) were explored. Visualising the nine scenarios in an isopleth resulted in overlapping areas and scenarios could not be identified separately. This was solved by combining all different scenarios in a compound symbol (point symbol) and chart (diagram) for each of the municipalities. As a result, information regarding differences within the municipality got lost.

Using the technique of map pair presentation climate and land use change effect could be separated into two maps. Now only three classes per map had to be visualised and texture could be used as an eligible graphic value (instead of value) to identify all scenarios separately.

A map series presentation was used to visualise all nine scenarios without introducing a threshold. The amount of nights in which the temperature rose above 20 °C in current climate ranged from 0 to 10 nights. However, in one of the climate scenarios (W+, see Fig. 4), the amount of nights ranged from 10 to 34 nights. So combining different climate scenarios in one legend with the same class interval increased the amount of classes. Normally five to seven classes for a choropleth map are suggested. Isopleth maps, or choropleth maps with very regular spatial patterns, can safely use more data classes because similar colours are seen next to each other, making them easier to distinguish (Slocum et al. 2009). Making use of diverging colour hue schemes, the number of classes can be extended; however, this might suggest a tipping point. Instead, a combination of value and saturation might be used to create a sequential enlarged colour hue scale. We used both to assess the effectiveness and visual preference.

**Fig. 5:**
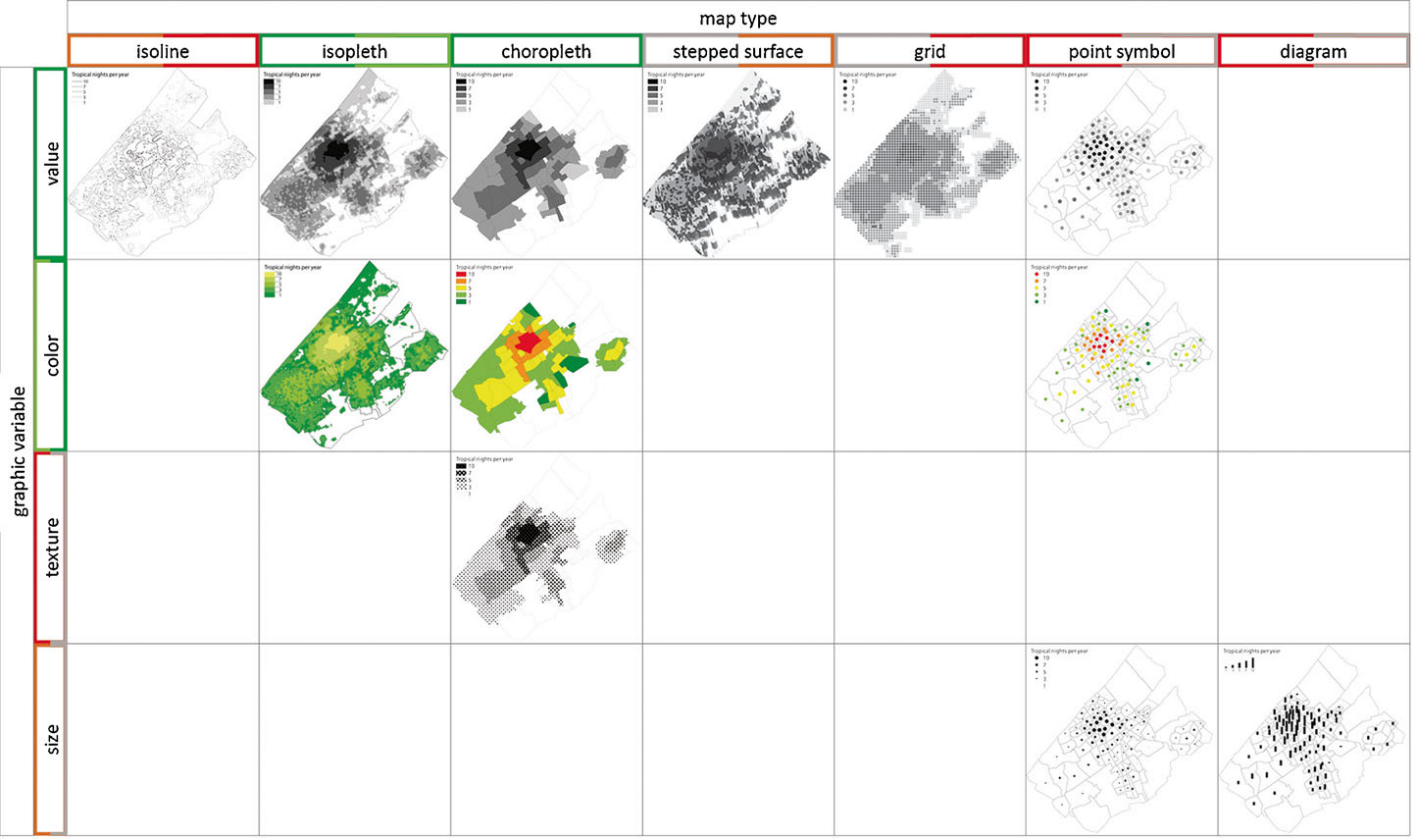
Overview of composed UHI maps for the Haaglanden region as a combination of map types and graphic variables for a single scenario. The colour of the borders indicates whether the map was preferred by users (*green* = most preferred; *red* = least preferred). The *left side* indicate the student results. The *right side* the results of the policy makers assessment. If *grey*, the map was not evaluated (mapping techniques that were least preferred in the student survey were excluded in the policy maker survey (i.e. map type: point symbol and diagram; graphic variable: texture and size; map presentation: bivariate and map pair). Instead, two new map types (grid and stepped surfaced) were added in the policy-maker survey)

**Fig. 6:**
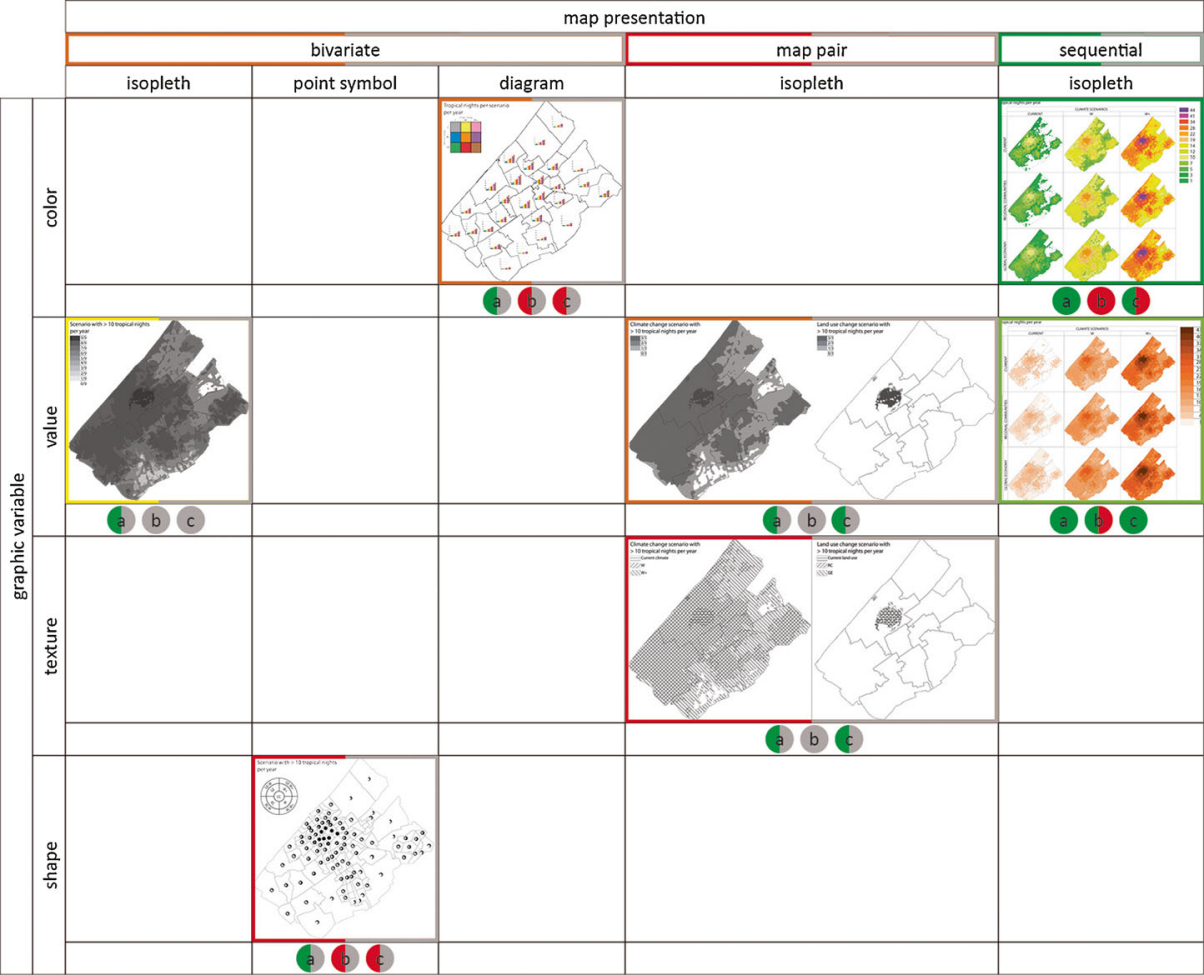
Overview of composed urban heat island maps for the Haaglanden region as a combination of map types, graphic variables and map presentation for multiple scenarios. The colour of the borders indicates whether the map was preferred by users (*green* = most preferred; *red* = least preferred). The colour of the dots beneath each map (*green* = effective; *red* = not effective) indicates whether tasks were performed correctly *a* depicting vulnerable area in different scenarios, *b* depicting the amount of nights and *c* depicting differences between climate and land use change separately. The *left side* of both border and dot indicate the student result. The *right side* the results of the policy makers. If *grey*, the map was not evaluated mapping techniques that were least preferred in the student survey were excluded in the policy maker survey (i.e. map type: point symbol and diagram; graphic variable: texture and size; map presentation: bivariate and map pair). Instead, two new map types (grid and stepped surfaced) were added in the policy-maker survey)

Figures 5 and 6 give an overview of the designed maps as discussed in the previous section. The maps were tested on their usability via an online survey as discussed in the next section.

To evaluate the usability of the different maps we performed a usability assessment via an online survey. Usability assessment is a technique used in user-centred interaction design to evaluate a prototype by testing it on real users (Faulkner 2000). Aim of the survey was to systematically measure visual preferences and effectiveness of the different mapping techniques. Visual preference was measured via pairwise comparisons. Questions regarding effectiveness were centred on three tasks: (a) depicting vulnerable area in different scenarios, (b) depicting differences between climate and land use change separately and (c) depicting the amount of nights. To increase the validity, the survey included two control questions and questions were randomised in two separate surveys. The survey includes questions about age, map reading familiarity and educational/professional background. The survey was first applied on students of the Wageningen University educated in the field of geo-visualisation to narrow the selection of mapping techniques for the policy-maker survey.

In the student survey, five map types (isoline, choropleth, isopleth, point symbol and diagram), four graphic variables (colour hue, value, texture and size) and three map presentations (bivariate, map pair and map series) were evaluated for satisfaction via 15 pairwise comparisons. The effectiveness was measured by 18 questions determining whether the information given by de maps was perceived correctly. Fifty students completed the student survey. In all comparisons, regarding map types, choropleth and isopleth were preferred above diagram, point symbol and isoline. Preferences between choropleth and isopleth were not discriminated. For the single scenario map, the point symbol type was preferred above a chart, but for the multiple scenario map, the majority preferred a chart above point symbols. A same difference for single and multiple scenarios goes for graphic value preferences. For the single scenario map, value (96 %) was preferable above colour hue (4 %), but for the multiple scenarios, map colour hue (52 %) and value (48 %) scored equally. Value and colour hue both were higher ranked than texture and size variables. A bivariate map presentation was preferred to a map pair presentation, but a map series was preferable above a bivariate presentation. All map techniques proved to be effective to correctly depict the most vulnerable area (average score of 92 %). To depict differences between climate and land use change separately, an isopleth map pair (texture) or map series (both value and colour hue) presentation was most effective (more than 70 % correctly answered). To depict the amount of nights, only the isopleth map series value had a score of more than 70 %.

Based on the results of the student survey, a selection of mapping techniques for the policy-maker survey was made. The mapping techniques were evaluated by six pairwise comparisons, regarding visual preferences of map types and graphic value of both single and multiple scenario maps. The map type point symbol and diagram were not evaluated in the policy-maker survey since they had the lowest preference and effectiveness score in the student survey. Instead, grid and stepped surface were introduced as new map types. Graphic variables were limited to value and colour hue since these two had the highest score regardless the map type. Map presentation was limited to a map series presentation for the same reason. Ten policy makers participated in the survey. The policy-maker survey resulted, similar to the student survey, in a shift of graphic value preference between single and multiple scenarios maps. Policy makers scored value and colour hue equally in the single scenario map and had a clear preference (8 out of 10) for colour hue in the multiple scenarios map. Again, isopleth and choropleth were the most preferred map types. Policy makers had a clear preference (7 of 10) for choropleth instead of isopleth, since it clearly indicates the impact for their neighbourhoods. Effectiveness was only measured for isopleth value and isopleth colour hue (both presented as map series for all nine scenarios). The majority of the respondents correctly indicated the most vulnerable area. To depict differences between climate and land use changes, only the value map proved to be effective. In contrast to the student results, none of the maps was effective to depict the amount of nights.

The final evaluation with the policy makers in the end stage of the UHI project was also used to determine how the visualisation of the constructed indicator supports the adaptation planning process. Policy makers were asked to comment on the map and give suggestions for further improvement. The value isopleth map series, which proved most effective by the survey, was included in an adaptation strategy report to further support the adaptation process. This report will be used by the policy makers of the involved governmental organisations, were decisions regarding adaptation measures at the local level will take place. To further support these organisations, also an interactive atlas was made. The atlas visualises the constructed UHI maps together with climate impacts as flooding and drought and sensitive objects into dynamic data displays. In this way, the problem of depicting the amount of nights as mentioned during the evaluation could be solved. Each scenario was displayed by a single display. Differences between the scenarios could be seen by rolling over scenario buttons. Due to the single display, the display size of the individual scenarios could be enlarged, making it easier to depict individual values. This could be further improved by visualising values while rolling over.

As already stated by Bertin (1983) quantitative measurement scales (ordinal, interval and ratio) should be best displayed by value. Based on the assessment we conclude that indeed, value proved to be more effective than colour hue. However, both students and policy makers preferred colour hue for multiple scenario presentations. Some studies point at the dominance of attractiveness over effectiveness and efficiency. Such an affective appraisal may have a serious impact on the understanding of the visualisation (Van Lammeren et al. 2010). Bertin also describes the concept of visual isolation (sélection), which suggests a maximum of eight classes to perceive differences. Our map series presentation contains 13 classes, which indeed makes it hard to depict the number of nights. However, by reducing the amount of classes, the minor difference between the land use scenarios would not be visible anymore. Identifying the task a map should serve is therefore important to select the number of classes. For this case, revealing the differences between the scenarios was found to be more important than depicting the exact number of nights.

## Reflection on methodology

**Fig. 7:**
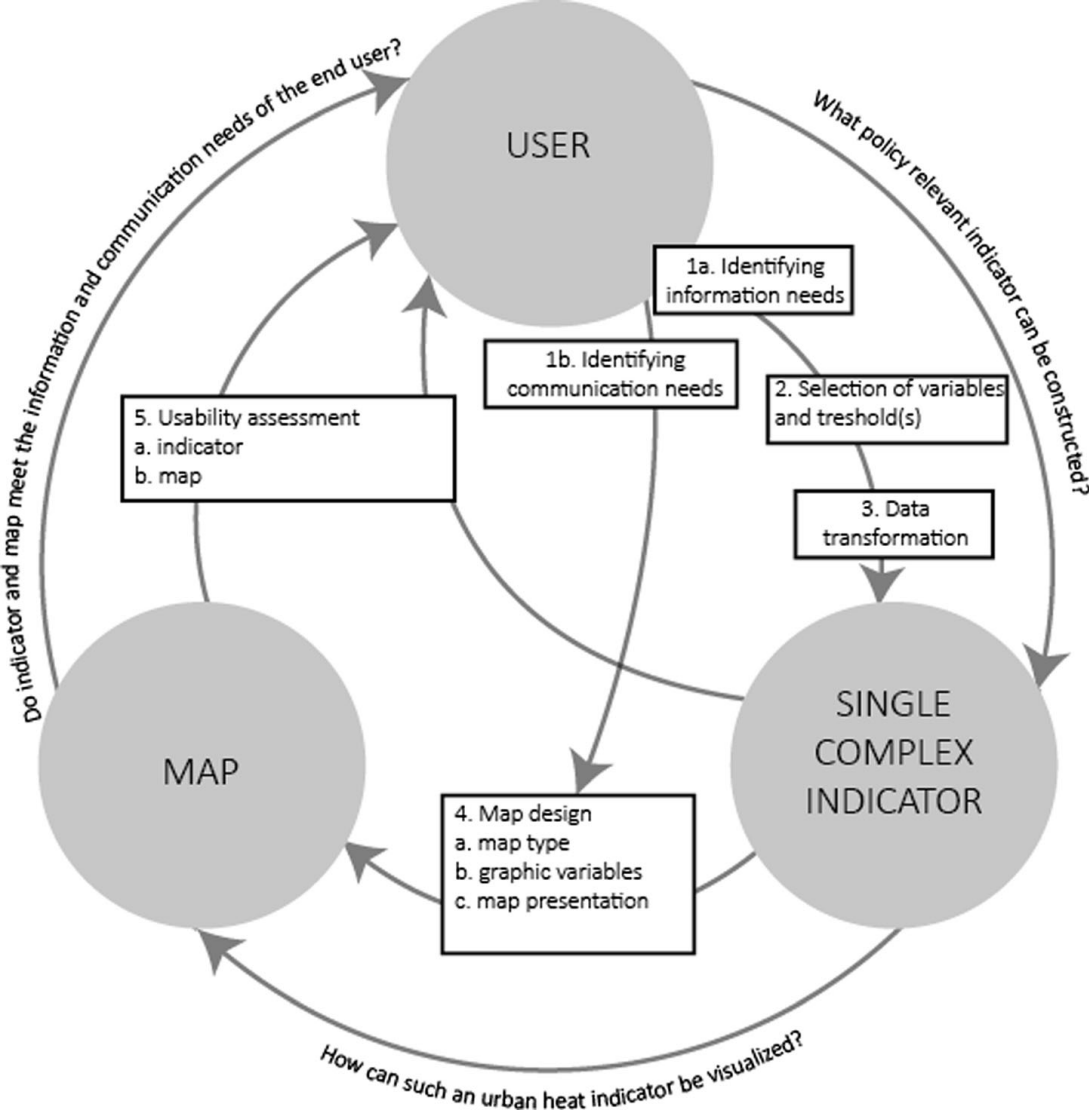
The information enrichment chain approach to support information transfer for adaptation planning. The information enrichment chain contains five steps that centralises the interaction between user, indicator and map. First, a single complex indicator to indicate the urban heat effect for local climate adaptation planning processes is defined based on information needs (*1a*), selection of variables and thresholds (*2*) and data transformation (*3*). Next, a map is designed based on the constructed indicator, communication needs (*1b*) and cartographic techniques (*4*). Finally, both indicator (*5a*) and maps (*5b*) are tested to evaluate whether they do meet user needs

van der Sandt et al. (2014) found out that at the local scale indicators should be developed in close collaboration with policy makers of the municipalities involved. The first step of our approach in which user needs were identified in advance proved important to select a policy-relevant indicator and variables. It also created an iterative process of knowledge exchange between scientists and policy. Next, transforming and visualising these variables in a single indicator and single map presentation proved effective to support the adaptation planning process. The presented methodology of selecting, transforming, combining, visualising and assessing increased the information density. Information was enriched without becoming too complex to grasp. Based on these findings, we introduce the information enrichment chain (see Fig. 7) as a stepwise approach to develop a single complex indicator to map climate impacts for local adaptation planning processes. The information enrichment chain contains five steps that centralises the interaction between actors, indicator and map presentation. First, a single complex indicator to indicate the urban heat effect for local climate adaptation planning processes is defined based on information needs (1a), selection of variables and thresholds (2) and data transformation (3). Next, a map is designed based on the constructed indicator, communication needs (1b) and cartographic techniques (4). Finally, both indicator (5a) and map (5b) are tested to evaluate whether they do meet user needs.

Identifying information needs from user (1a) is needed to determine the core variables of interest and the derived indicators to discuss effects and sensitivity: What do your users want to know about the urban heat island potential and which indicators can be derived from that need?

Identifying communication needs from user (1b) centres the message, goal and outcome of the final visualisation: e.g. Is the visualisation meant to inspire or to underpin the policy making process?

Based on the information needs, the different elements of the indicator as variables and thresholds can be selected (2). The selection of variables and thresholds also depends on data availability.

When the selection of the variables has taken place, data can be collected and transformed to construct the indicator (3). For each variable, the underlying data and the methods to transform the data should be identified. Therefore, insight in the underlying data and formulas is needed. In this stage, also the use of weighing factors to combine the variables in one indicator should be discussed.

Within each step of the enrichment chain as described above, the amount of variables taken into account increases. Visualising this information should follow a design approach in which different cartographic techniques like map types, graphic variables and map presentations are explored (4). The most effective map design depends on both information and communication needs. Therefore, the entire visualisation process of a climate indicator cannot be fully automated. Expert assessment is needed to convert model outcomes into user-friendly maps and to validate the usability of these maps.

Finally, both the indicator and map should be evaluated by a panel of end users to find out whether this is an effective and usable way of visualising the effects of climate change and land use change (5).

## Discussion

This paper aimed at translating scientific information on urban heat in terms of a policy-relevant indicator and map presentation. The literature review on urban heat mapping revealed a wide range of indicators to indicate the urban heat impact. Even a conclusive indicator for temperature differences could not be defined. We limited urban heat to nocturnal air temperature, and we introduced a threshold to better indicate the urban heat impact. It can be argued that effective temperature (ET) and apparent temperature (AT) (Blazejczyk et al. 2012) give a better assessment of the thermal exposure on the human body. Other climatic circumstances might require other thresholds.

Similar with the conclusions of Alcoforado and Andrade (2008), only one of the analysed papers in our literature review mentioned the impacts of global warming on the UHI and none of them succeeded to map future impacts. In our method, we have used the KNMI climate scenario’s to cascade the impacts of global warming into regional changes. Tailoring global scenarios is needed to reveal enough spatial detail (Van den Hurk et al. 2006).

With respect to the mapping techniques, both the literature review and our case study were limited to static displays. Besides static displays, uncertainties can also be revealed by dynamic visualisation techniques like animated map ensembles (Opach et al. 2014). As time is intrinsic to the form of an animation, the suggestion of changes through time and space might be more effective (DiBiase et al. 1992; Resch et al. 2013). Dynamic mapping also allow users to modify the data display (DiBiase et al. 1992), which might improve legibility.

Although policy makers had a preference for the colour hue map, using a red-green colour scale might be an obvious but not the best idea as it may exclude people with colour deficiencies. Testing colour deficiencies was not part of the survey but should be taken into account for future map design.

## Conclusions

Aim of this paper was to translate scientific information on urban heat into policy-relevant and usable information to support local adaptation planning processes. The first question addressed was to construct a policy-relevant urban heat indicator for local climate adaptation planning processes. Literature analysis did not reveal a dominant policy relevant urban heat indicator for planning purposes. None of the reviewed articles integrated information on land use and climate changes impacts or included thresholds to determine the impact. The case study illustrated that tailoring and combining global information with local information was needed to set the agenda for adaptation planning at the local scale. Transforming this information into a single complex indicator provided accessible and relevant high dense information. Defining the different components of the indicator together with policy makers improved the information exchange between science and policy and provides a basis for the development and application of sustainable adaptation strategies. As on global scale urban areas continue to expand, the method as presented in this paper can be used to translate scientific information of the urban heat island into urban design at a global scale.

The second question addressed was to investigate how such an urban heat indicator could be visualised. Our findings clearly reveal that it was important for policy makers to summarise future impact of climate and land use change in one single map presentation. The case study illustrated that traditional cartographic visualisations were adequate to visualise climate impact information. For the Haaglanden region, a map series presentation proved to be effective to visualise complex information in a single image, although adding interactive techniques might improve legibility.

Finally, the methodology of this study entitled the information enrichment chain seems a promising approach for translating outcomes of climate science impact studies into policy-relevant and usable information to support local adaptation planning processes. We suggest to test whether the information enrichment chain approach can be used for other climate events, like flooding and drought.
